# Computation of the normalized cross-correlation by fast Fourier transform

**DOI:** 10.1371/journal.pone.0203434

**Published:** 2018-09-20

**Authors:** Artan Kaso

**Affiliations:** Department of Diagnostic Radiology and Nuclear Medicine, University of Maryland, Baltimore, MD, United States of America; University of North Carolina at Chapel Hill, UNITED STATES

## Abstract

The normalized cross-correlation (NCC), usually its 2D version, is routinely encountered in template matching algorithms, such as in facial recognition, motion-tracking, registration in medical imaging, etc. Its rapid computation becomes critical in time sensitive applications. Here I develop a scheme for the computation of NCC by fast Fourier transform that can favorably compare for speed efficiency with other existing techniques and may outperform some of them given an appropriate search scenario.

## Introduction

Covariance, by definition, provides a measure of the strength of the correlation between two sets of numbers (or time series). A serious setback of the covariance is its dependence on the amplitude of either of the series that are compared. This dependency is eliminated if one uses the normalized form of the covariance, referred to as the normalized cross-correlation (otherwise known as the correlation coefficient). The simplest form of the normalized cross-correlation (NCC) is the cosine of the angle *θ* between two vectors **a** and **b**:

$$\begin{array}{c} \text{NCC}=\cos\theta =\frac{a\cdot b}{\left|a||b\right|}=\frac{\sum_{i}a_{i}b_{i}}{\sqrt{\sum_{i}a_{i}^{2}}\sqrt{\sum_{i}b_{i}^{2}}},\;-1\leq \text{NCC}\leq 1. \end{array}$$ (1)

NCC is one of those quantities with application in a variety of research fields as diverse as physics [1, 2], signal processing [3–7], engineering [8, 9], medical imaging [10], and statistical finance [11].

The similarity of the mathematical expression for the numerator of the NCC (see section 1, Eq (4)) with that of a convolution is striking and implicates that its computation must be optimal in the Fourier transform space [3]. Starting from this premise, Lewis [5] conjectured and numerically proved that the numerator of the NCC can be efficiently computed in either the spatial or the frequency domain contingent upon the parameters of the problem. For an overall rapid computation of the NCC it is necessary to have a likewise efficient computation of its denominator.

A number of techniques have been proposed by different authors for the fast computation of the NCC. These techniques have their merits and drawbacks, most of them excelling in very specialized cases. Here we mention three of these techniques: [5, 6] and [10].

Lewis [5] proposes the computation of the denominator of the NCC in direct space by keeping sum-tables in order to avoid repetitive computations. The numerator of the NCC can however be computed either in the direct space or in the frequency domain. As Lewis notes in the introduction to [5] “Unfortunately the normalized form of correlation does not have a correspondingly simple and efficient frequency domain expression”. This statement is of importance because only one or the other (spatial or frequency domain) computation can be optimally fast given the parameters of an application. This fact is duly observed by Lewis in section 4 of [5] where he writes, referring to searching a feature of length *N* in a time series of length *M*:

When M is much larger than N the complexity of direct ‘spatial’ computation is approximately *N*^2^ *M*^2^ multiplications/additions and the direct method is faster than the transform method. The transform method becomes relatively more efficient as *N* approaches *M* and with larger *M*, *N*.

The computation of the denominator in the frequency domain is addressed in the present paper. While the numerator of the NCC is the covariance between two different time series, either of the two terms under the square root in the denominator represents the covariance of that series with itself (i.e. auto-covariance) at lag zero. If an efficient computation for the numerator in the frequency space is possible, one can expect this to be possible for the denominator as well.

Luo and Konofagou [10] propose the computation of the NCC exclusively in direct space by keeping sum-tables for both the numerator and the denominator. While Luo’s approach cannot outperform Lewis computation when a single feature of a given size, selected from one time series, is exhaustively searched for throughout the other time series, it can however be advantageous for other search scenarios [10].

Yoo and Han [6] propose a scheme for the computation of the NCC that considerably reduces both the number and the complexity of the required computational operations as compared with other full computing schemes. The proposed scheme is based on approximative assumptions. The gain in the computational speed is done at the expense of an algorithm that generates false positives in a feature search scenario. The rate of false positives depends on the noise level present in the time series, and can be minimized by tuning parameters in the algorithm. This performance dependence on the noise level can be considered a setback when “shoot first and ask later” approach can be a problem. It is also not clear how this algorithm can be extended and will perform if signals were complex-valued.

The algorithm presented in this paper can handle complex-value signals. It can be considered as an extension/supplement of the work done by Lewis in that the fast Fourier transform (FFT) is used to compute both the numerator and the denominator of the NCC. The proposed algorithm opens the possibility for the computational parallelization (FFT threads) of the procedure and may offer speed gains for appropriate template and search region size [5].

The paper is organized as follows. Section 1 exposes the mathematical formula for the 1D complex NCC and lays the ground for developing the proposed FFT computational scheme, which is done in Section 2. The 2D formulas can be found in Appendix C, their derivation follows exactly the same steps as the 1D. In Results, two measurements are considered as test cases. Both of them are real-valued, the first is 1D and the other 2D. The python code developed for the computation of the NCC can handle complex-value measurements and is listed in Appendix B. An illustrative complex valued 1D test case is provided in Supplements 1. Python programs as well as the data sets used for the 1D and 2D illustrations can be found in the supplements.

The time spent in computing the NCC for the 1D test case is tabulated below for several numeralical methods/schemes used. The timing is done on optimized C++ code, it certainly is processor dependent and is shown here to create a general comparative idea. The execution time is averaged over 15 runs, with 5 ms wait time between runs to account for the processor delays. The parameter values used for this 1D test can be found in the Results section. The test shows that:

1. Lewis NCC with direct space computation of the numerator is slightly slower than when FFT is used instead (the denominator is computed in direct space using sum-tables in both cases), Table 1.
2. The proposed computation is slower than Lewis method with FFT computation of the numerator for the parameter values, i.e. template and search region lengths, investigated in this paper, Table 2. An additional test case comprising 1D complex value measurements and of larger size is investigated in Supplement 1. Other possible computational optimizations (multi-threads, wisdom FFT) are not investigated in this work.
3. The Luo’s computation of NCC using sum-tables for the numerator is not faster than the Lewis algorithm for the search scenario tested here, Table 3.

**Table 1:** The time expendend in the computation of the NCC according to Lewis algorithm [5] when two approaches are used.

	mean time (*μ*s)	std
Lewis, numerator direct space	99.40	0.01
Lewis, numerator frequency space	26.70	0.01

**Table 2:** The time expendend in the computation of the NCC according to Lewis algorithm [5] and the algorithm proposed in this paper.

	mean time (*μ*s)	std
Lewis, numerator frequency space	26.70	0.01
Proposed method	38.20	0.05

**Table 3:** The time expendend in the computation of the NCC according to Lewis algorithm [5] and the algorithm proposed by Luo [10].

	mean time (*μ*s)	std
Lewis, numerator frequency space	26.70	0.01
Luo’s method, real	241.20	0.09
Luo’s method, complex	463.93	12.97

To have a fair comparison of the computational speed between the Lewis’ and the proposed method, both methods are implemented for complex-value time series (requiring approximately twice as many computations as for real-value time series, see the above listed computational times of Luo’s method that handle real or complex time series). The two methods are then however applied on real-value time series for simplicity.

### Section 1

Consider two, complex-value and periodic, functions of time: *f*(*t*) and *g*(*t*), both having the same time period *T*. Suppose that these two functions are uniformly sampled with a time step Δ*t* such that *T* = *n*Δ*t*. Using the discrete Fourier transform formalism the sampled function *f*(*t*_*i*_) ≡ *f*_*i*_, where *t*_*i*_ ≡ *i*Δ*t*, 0 ≤ *i* < *n*, can be uniquely described by a complementary set of complex-values {*α*_*k*_}

$$\begin{array}{cc} f_{i}= & \frac{1}{n}\sum_{k=0}^{n-1}\alpha_{k}\;e{\;}^{j2\pi \frac{ki}{n}}\;0\leq i<n\\ & \Leftrightarrow \left\{f_{i}\right\}=if\;ft\left(\left\{\alpha_{k}\right\}\right) \end{array}$$ (2)

$$\begin{array}{cc} \alpha_{k}= & \sum_{i=0}^{n-1}f_{i}\;e{\;}^{-j2\pi \frac{ki}{n}}\;0\leq k<n\\ & \Leftrightarrow \left\{\alpha_{k}\right\}=f\;ft\left(\left\{f_{i}\right\}\right) \end{array}$$ (3)

Here *fft*, *ifft* are respectively the fast Fourier transform function and its inverse as defined in MATLAB, *j* represents the imaginary unit, *i*, *k*, *p*, *q* will represent integer variables, and *n*, *m* are integer parameters. Similarly, we symbolically write

$$\left\{g_{i}\right\}=if\;ft(\left\{\beta_{k}\right\})$$

$$\left\{\beta_{k}\right\}=f\;ft(\left\{g_{i}\right\})$$

**Fig 1:**
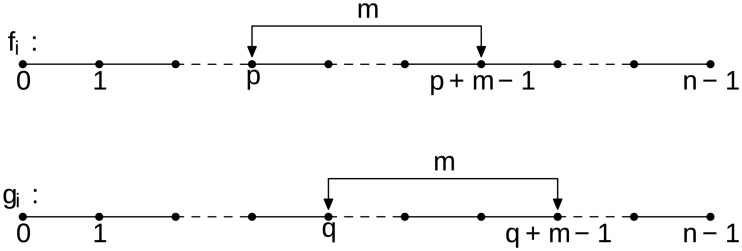
Schematic of two subsets of samples of length *m* drawn from the T-periodic time series *f*(*t*) and *g*(*t*), uniformly sampled with *n* samples per period.

Consider two subsets of consecutive data points of the same length *m* extracted from the discrete time series {*f*_*i*_} and {*g*_*i*_}, as shown in Fig 1. Hereafter *p*, *q* ∈ [0, *n* − *m* + 1].

The normalized cross-correlation (NCC) between these two subsets is defined as

$$\begin{array}{ll} \text{NCC} & =\\ & \frac{\frac{1}{m}\sum_{i=p}^{p+m-1}{\left(f_{i}-\bar{f}\right)}^{*}\cdot \;\left(g_{i+q-p}-\bar{g}\right)}{\sqrt{\frac{1}{m}\sum_{i=p}^{p+m-1}{\left|f_{i}-\bar{f}\right|}^{2}}\sqrt{\frac{1}{m}\sum_{i=q}^{q+m-1}{\left|g_{i}-\bar{g}\right|}^{2}}} \end{array}$$ (4)

where $\bar{f}\equiv \frac{1}{m}\sum_{i=p}^{p+m-1}f_{i}$ and $\bar{g}\equiv \frac{1}{m}\sum_{i=p}^{p+m-1}g_{i+q-p}=\frac{1}{m}\sum_{i=q}^{q+m-1}g_{i}$. Introducing the notations $\bar{\left|f\right|{\;}^{2}}\equiv \frac{1}{m}\Sigma_{i=p}^{p+m-1}{\left|f_{i}\right|}^{2}$, $\bar{\left|g\right|{\;}^{2}}\equiv \frac{1}{m}\Sigma_{i=q}^{q+m-1}{\left|g_{i}\right|}^{2}$, and $\bar{f^{*}g}\equiv \frac{1}{m}\sum_{i=p}^{p+m-1}f_{i}^{*}g_{i+q-p}$ it can be rewritten as

$$\begin{array}{c} \text{NCC}=\frac{\bar{f^{*}g}-{\left(\bar{f}\right)}^{*}\bar{g}}{\sqrt{\bar{{\left|f\right|}^{2}}-{\left|\bar{f}\right|}^{2}}\sqrt{\bar{{\left|g\right|}^{2}}-{\left|\bar{g}\right|}^{2}}} \end{array}$$ (5)

In the following section I present the scheme for computing the NCC using the fast Fourier transform.

## Section 2

Start by calculating

$$\begin{array}{ll} \bar{f} & =\frac{1}{m}\sum_{i=p}^{p+m-1}f_{i}=\frac{1}{n}\sum_{k=0}^{n-1}\alpha_{k}\frac{1}{m}\sum_{i=p}^{p+m-1}e{\;}^{j2\pi \frac{ki}{n}}\\ & =\frac{1}{n}\sum_{k=0}^{n-1}\alpha_{k}\;e{\;}^{j2\pi \frac{kp}{n}}\frac{1}{m}\sum_{i=0}^{m-1}e{\;}^{j2\pi \frac{ki}{n}}\\ & \equiv \frac{1}{n}\sum_{k=0}^{n-1}\alpha_{k}\gamma_{k}\;e{\;}^{j2\pi \frac{kp}{n}}=if\;ft\left(\left\{\alpha_{k}\gamma_{k}\right\}\right){|}_{p} \end{array}$$ (6)

where

$$\begin{array}{c} \gamma_{k}\equiv \frac{1}{m}\sum_{i=0}^{m-1}e{\;}^{j2\pi \frac{ki}{n}}=\frac{1}{m}\frac{e{\;}^{j2\pi \frac{km}{n}}-1}{e{\;}^{j2\pi \frac{k}{n}}-1} \end{array}$$ (7)

is the Dirichlet kernel. Next calculate

$$\begin{array}{ll} \bar{{\left|f\right|}^{2}} & =\frac{1}{m}\sum_{i=p}^{p+m-1}{\left|f_{i}\right|}^{2}\\ & =\frac{1}{m}\sum_{i=p}^{p+m-1}\frac{1}{n}\sum_{k=0}^{n-1}\alpha_{k}^{*}{\text{e}}^{-j2\pi \frac{ki}{n}}\frac{1}{n}\sum_{k^{\prime }=0}^{n-1}\alpha_{k^{\prime }}{\text{e}}^{j2\pi \frac{k^{\prime }i}{n}}\\ & =\frac{1}{n^{2}}\sum_{k,k^{\prime }=0}^{n-1}\alpha_{k}^{*}\alpha_{k^{\prime }}{\text{e}}^{j2\pi \frac{\left(k^{\prime }-k\right)p}{n}}\frac{1}{m}\sum_{i=0}^{m-1}{\text{e}}^{j2\pi \frac{\left(k^{\prime }-k\right)i}{n}} \end{array}$$ (8)

By variable substitution *k* = *n* − 1 − *k*″ and relabeling obtain

$$\begin{array}{ll} \bar{{\left|f\right|}^{2}} & =\frac{1}{n^{2}}\sum_{k,k^{\prime }=0}^{n-1}\alpha_{n-1-k}^{*}\alpha_{k^{\prime }}{\text{e}}^{j2\pi \frac{\left(k+k^{\prime }+1\right)p}{n}}\\ & \left(\frac{1}{m}\sum_{i=0}^{m-1}{\text{e}}^{j2\pi \frac{\left(k+k^{\prime }+1\right)i}{n}}\right)\\ & \equiv \frac{1}{n^{2}}\sum_{k,k^{\prime }=0}^{n-1}a_{k,k^{\prime }}{\text{e}}^{j2\pi \frac{\left(k+k^{\prime }+1\right)p}{n}} \end{array}$$ (9)

where

$$\begin{array}{c} a_{k,k^{\prime }}\equiv \alpha_{n-1-k}^{*}\alpha_{k^{\prime }}\frac{1}{m}\sum_{i=0}^{m-1}e{\;}^{j2\pi \frac{(k+k^{\prime }+1)i}{n}} \end{array}$$ (10)

**Fig 2:**
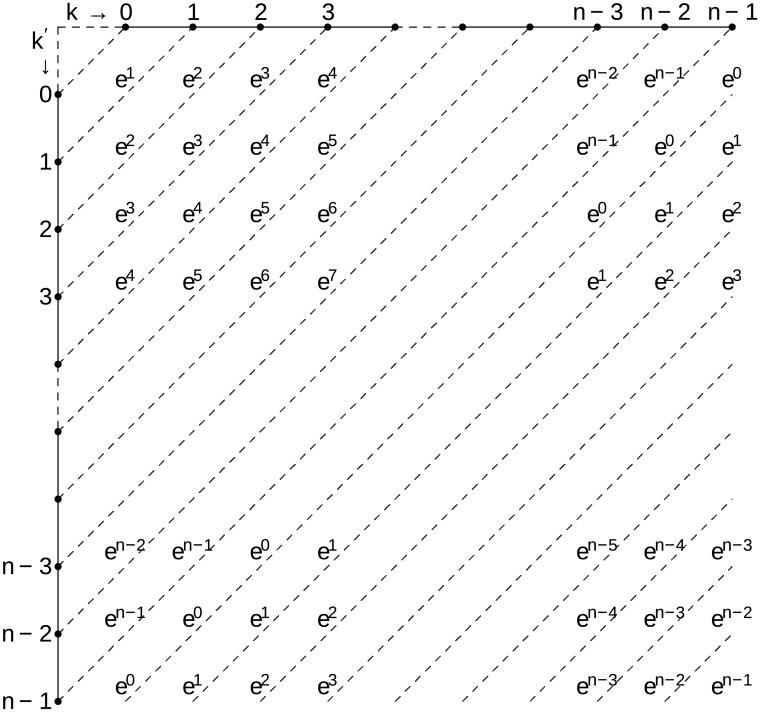
Sketch visualizing the transformation of a double sum performed along the columns then along the rows, to a sum of the sums performed along the secondary diagonals.

The dependency of *a*_*k*, *k*′_ on *m* is left out of notation for clarity. In the following I transform the double sum to a single sum. Fig 2 helps with visualizing the procedure.

The double sum suggests that we sum along columns per each row, and then sum all terms so obtained. Chose, instead, to first sum along the secondary diagonals per each secondary diagonal, and then sum all obtained terms. By doing this the *k* + *k*′ + 1 = *l* mod *n* remains constant during the first summation and the exponential term depending on *p* is factorized. Therefore

$$\begin{array}{c} \bar{{\left|f\right|}^{2}}=\frac{1}{n}\sum_{l=0}^{n-1}\zeta_{l}\;{\text{e}}^{j2\pi \frac{lp}{n}}=ifft\left(\left\{\zeta_{l}\right\}\right){|}_{p} \end{array}$$ (11)

with

$$\begin{array}{c} \zeta_{l}=\{\begin{array}{cc} \frac{1}{n}\sum_{k=0}^{n-1}a_{k,n-1-k} & l=0\\ \frac{1}{n}(\sum_{k=0}^{l-1}a_{k,l-1-k}\;+\\ \;\sum_{k=l}^{n-1}a_{k,n+l-1-k}) & 1\leq l<n \end{array} \end{array}$$

Straightforward calculations using definitions and index relabeling reveal that

$$\begin{array}{c} \sum_{k=0}^{n-1}a_{k,n-1-k}=\sum_{k=0}^{n-1}\alpha_{k}^{*}\alpha_{k} \end{array}$$ (12)

$$\begin{array}{ll} & \left(\sum_{k=0}^{l-1}a_{k,l-1-k}+\sum_{k=l}^{n-1}a_{k,n+l-1-k}\right)\\ & =\gamma_{l}\frac{1}{n}(\sum_{k=0}^{n-l-1}\alpha_{k}^{*}\alpha_{l+k}+\sum_{k=n-l}^{n-1}\alpha_{k}^{*}\alpha_{l+k-n}) \end{array}$$ (13)

Now,

$$\begin{array}{ll} \frac{1}{n}\sum_{k=0}^{n-1}\alpha_{k}^{*}\alpha_{k} & =\sum_{i=0}^{n-1}{\left|f\right|}_{i}^{2}\\ & =\sum_{i=0}^{n-1}{\left|f\right|}_{i}^{2}{\text{e}}^{-j2\pi \frac{li}{n}},\;l=0 \end{array}$$ (14)

$$\begin{array}{ll} & \sum_{k=0}^{n-l-1}\alpha_{k}^{*}\alpha_{l+k}\\ & =\sum_{i,i^{\prime }=0}^{n-1}f_{i}^{*}f_{i^{\prime }}\;e{\;}^{-j2\pi \frac{li^{\prime }}{n}}\sum_{k=0}^{n-l-1}e{\;}^{j2\pi \frac{k(i-i^{\prime })}{n}} \end{array}$$ (15)

$$\begin{array}{ll} & \sum_{k=n-l}^{n-1}\alpha_{k}^{*}\alpha_{l+k-n}\\ & =\sum_{i,i^{\prime }=0}^{n-1}f_{i}^{*}f_{i^{\prime }}\;e{\;}^{-j2\pi \frac{li^{\prime }}{n}}\sum_{k=n-l}^{n-l}e{\;}^{j2\pi \frac{k(i-i^{\prime })}{n}} \end{array}$$ (16)

therefore

$$\begin{array}{l} \frac{1}{n}\left(\sum_{k=0}^{n-l-1}\alpha_{k}^{*}\alpha_{l+k}+\sum_{k=n-1}^{n-l}\alpha_{k}^{*}\alpha_{l+k-n}\right)\\ =\sum_{i=0}^{n-1}{\left|f\right|}_{i}^{2}{\text{e}}^{-j2\pi \frac{li}{n}},\;1\leq l<n \end{array}$$ (17)

Relabeling, noting that *γ*_0_ = 1, and condensing

$$\begin{array}{ll} \zeta_{k} & =\gamma_{k}\sum_{i=0}^{n-1}{\left|f\right|}_{i}^{2}{\text{e}}^{-j2\pi \frac{ki}{n}},\;0\leq k<n\\ & =fft\left(\left\{{\left|f\right|}_{i}^{2}\right\}\right){|}_{k}\gamma_{k} \end{array}$$ (18)

Then

$$\begin{array}{c} \bar{{\left|f\right|}^{2}}=ifft\left(\left\{fft\left(\left\{{\left|f\right|}_{i}^{2}\right\}\right){|}_{k}\gamma_{k}\right\}\right){|}_{p} \end{array}$$ (19)

Similar calculations hold for $\bar{g}$ and $\bar{{\left|g\right|}^{2}}$.

Finally calculate the remaining term that completes the computation of NCC using the fast Fourier transform

$$\begin{array}{ll} & \bar{f^{*}g}=\frac{1}{m}\sum_{i=p}^{p+m-1}f_{i}^{*}g_{i+q-p}=\frac{1}{m}\sum_{i=0}^{m-1}f_{i+p}^{*}g_{i+q}\\ & =\frac{1}{n}\sum_{k=0}^{n-1}\alpha_{k}^{*}(\frac{1}{m}\sum_{i=0}^{m-1}g_{i+q}\;e{\;}^{-j2\pi \frac{ki}{n}})e{\;}^{-j2\pi \frac{kp}{n}}\\ & \equiv \sum_{k=0}^{n-1}\alpha_{k}^{*}\eta_{k}\;e{\;}^{-j2\pi \frac{kp}{n}}=f\;ft\left(\left\{\alpha_{k}^{*}\eta_{k}\right\}\right){|}_{p} \end{array}$$ (20)

where

$$\begin{array}{ll} \eta_{k} & =\sum_{i=0}^{m-1}\frac{g_{i+q}}{n\;m}\;e{\;}^{-j2\pi \frac{ki}{n}}\\ & \equiv \sum_{i=0}^{n-1}h_{i}\;e{\;}^{-j2\pi \frac{ki}{n}}=f\;ft\left(\left\{h_{i}\right\}\right){|}_{k} \end{array}$$ (21)

with

$$\begin{array}{c} h_{i}=\{\begin{array}{cc} g_{i+q}/\left(n\;m\right) & 0\leq i<m\\ 0 & m\leq i<n \end{array} \end{array}$$

Then

$$\begin{array}{c} \bar{f^{*}g}=f\;ft\left(\left\{\alpha_{k}^{*}f\;ft\left(\left\{h_{i}\right\}\right){|}_{k}\right\}\right){|}_{p} \end{array}$$ (22)

## Results

The method is illustrated with two examples, both drawn from MRI measurements.

**Fig 3:**
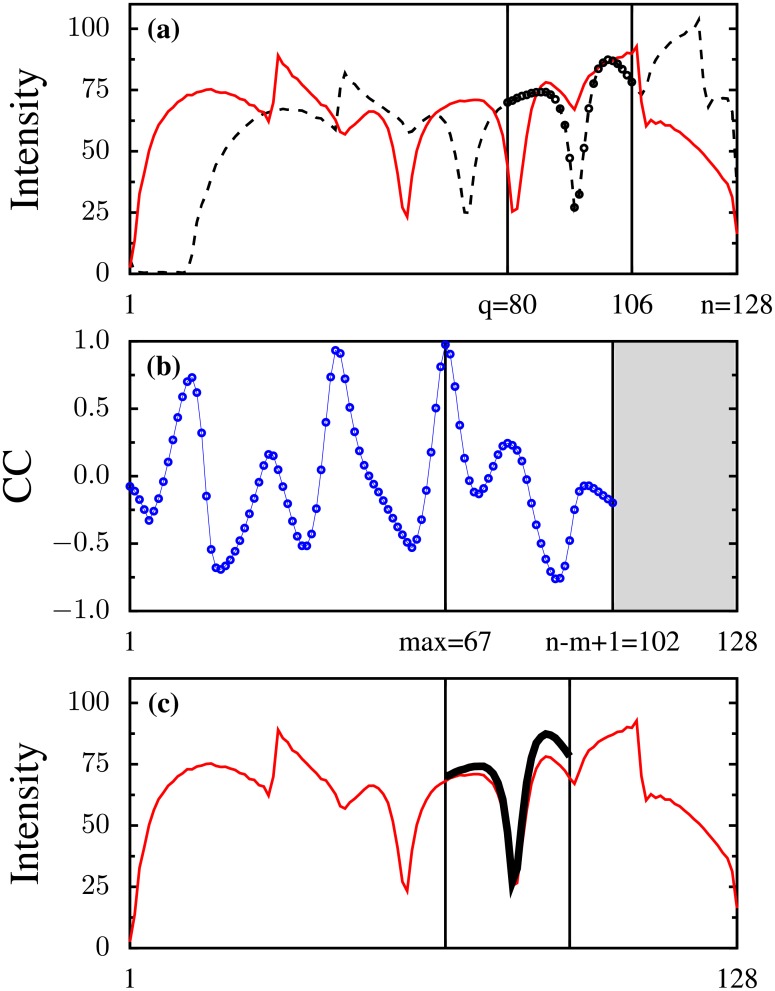
(**a**) Two intensity profiles, a template of *m* = 27 samples is selected on one of them (the dashed, black line). (**b**) The template is slid along the other profile (the solid, red line) and the normalized cross-correlation (NCC) is computed for each possible position. NCC is then plotted as a function of the position of the left extremity of the template. (**c**) The template is drawn on top of the most similar chunk.

The first example is 1D and relates to a rigid phantom, the intensity profile of which is measured at two different positions, 30 mm apart (as read on the landmark meter of the scanner) along the bore of the magnet. The two profiles differ partly because the static magnetic field in the scanner deviates even so slightly from homogeneity and partly because of the non-linearity of the gradients. The contribution of the measurement noise to the profiles’ difference is of a less consequence. In this measurement the field of view (FOV) is 300 mm and the resolution *xres* = 128. The intention is to find the phantom’s displacement by using only the information provided by these two intensity profiles. This is done by selecting a template, which represents a feature of interest, in one of the profiles, say the dashed, black line in Fig 3a. In our example the start position and the length of the selected template are *q* = 80 and *m* = 27 samples respectively. We then search for the presence of a similar feature in the other profile, the navigator, plotted as the solid, red line in Fig 3a. Because the two profiles are different, the degree of similarity between the template and chunks of the same size extracted exhaustively from the navigator profile is determined from the value of the normalized cross-correlation (NCC). The similarity of the chunk to the template is higher the larger the value of NCC between the two is, with the chunk being identical to the template for NCC = 1. The total number of chunks that can be construed from the navigator profile is *n* − *m* + 1. Here *n* = *xres* = 128, and *n* − *m* + 1 = 102. The NCC values are plotted in Fig 3b and show that the feature of interest is located at the start position *max* = 67 where NCC reaches it maximum value. Fig 3c shows how the template visually compares with the most similar chunk if drawn on top of it. From the difference between the start positions of the template and the most similar chunk we can compute the translational distance between the two intensity profiles as: *FOV* · (*q* − *max*)/*xres* ∼ 30 mm, in good agreement with what the landmark meter on the scanner displayed. A python version of the code generating the data used in the plots is listed in Appendix B and can be downloaded from the supplements.

**Fig 4:**
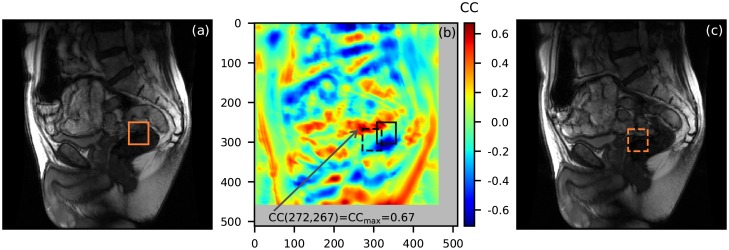
Two 2D images (a, c) taken at two different time points show the reconfiguration of the patient’s internals. (**a**) A template of size 48x54 is selected on the first image. (**b**) The template is slid along the second image and the normalized cross-correlation (NCC) is computed for every possible position. The NCC map is plotted as a function of the top left corner of the template’s position. The template region and the most similar chunk are plotted as the solid and the dashed square respectively. (**c**) The most similar chunk is drawn as the dashed square on the second image.

The second example is 2D and relates to a patient resting on the scanner’s table while MR images are being taken. There is no gross motion of the patient, but there is motion of his bowels. The intention is to track the motion of a region of interest from one time frame to the next. Here we display two time frames and select a template on the first. We search throughout the second frame to find where the most similar (with the template) 2D chunk is located. This test case is one of the most complicated for motion tracking as there is displacement of the region of interest out of the 2D fixed plane where images are being taken into the third dimension as well as nonrigid transformation, i.e. deformation, of the tissues. NCC is not the most suitable metric to be used for feature tracking in cases like this, at least not without any adaptation [7]. However the method performs reasonably well given that the bowels reconfigure considerably from one frame to the next, as evidenced by the low value (0.67 vs. 1) of the maximum NCC. The result is presented in Fig 4. A python version of the code used can be downloaded from the supplements. Motion tracking using the NCC would perform better the smaller the topological difference between consecutive time frames is. Unfortunately this is not always possible with MR Images in spatial domain: the spatial resolution of an MR image is directly related to the time spent collecting data for its reconstruction. If the topological difference between two consecutive time frames is large, motion tracking using the NCC can encounter a “glitch” where a chunk of irrelevant anatomy is selected as the most similar region with the template. This encounter will result either with a lost track or a return to the track after the abrupt excursion away from the track (hence the term “glitch”).

Comments in the listed code are kept to a minimum with the understanding that the code flow and variable labeling closely follows the derivations presented in the text of this paper. In the code, the computations related to the template are separated from other cross-correlation computations. This is done to increase the computational efficiency since the parameters related to the template need to be computed only once while the rest of NCC computation might need to be performed over a multitude of navigators (i.e. time frames). The code for computing the 1D NCC with Lewis’ [5], and Luo’s [10] algorithms is listed as well so that a comparison of the NCC values obtained with these methods can be done. The provided python code cannot however be used to compare the time efficiency of these numerical methods. Their time performance comparison is done by this author using optimized C++ code.

## Conclusion

In this paper I have demonstrated an algorithm for the computation of the normalized cross-correlation (NCC) using the FFT. It is shown that, for the data sets investigated, the computation of the NCC fully in the transform space is not faster than the optimized algorithm proposed by Lewis (in the Lewis optimized algorithm the numerator of the NCC is computed in the transform space and the denominator in the direct space). The comparative computational speed between the two algorithms seems to be unaffected by the template and the search window sizes with the Lewis’ algorithm always performing faster. This finding is somewhat of a surprise considering that the physical nature of the numerator and denominator in the NCC formula is almost “identical” with the numerator standing as the correlation between two time series (cross-correlation) and the denominator involving the correlation of a time series with itself (auto-correlation). A final comparison of the computational speed between these two algorithms (Lewis’ optimized algorithm and the one proposed here) would be their thread parallelization.

## Appendix A

Define the sum-table for the numerator of the NCC according to [10], as

$$\begin{array}{c} s_{i,d}=\{\begin{array}{cc} 0 & i=0\\ s_{i-1,d}+f_{i-1}^{*}g_{i-1+d} & 1\leq i<n+1 \end{array},\;0\leq d<n \end{array}$$

with *d* the lag between the template *f* and the navigator chunk *g*. I will use the *n*-periodicity of the navigator to keep the indexing in the chunk relevant. Denoting with *p*, *q* ∈ [0, *n* − *m* + 1] the positioning of the template and the navigator chunk from their respective time series origins, and using the definition of *s*_*i*,*d*_ above, we write

$$\begin{array}{ll} \sum_{i=0}^{m-1}f_{i+p}^{*}g_{i+q} & =\sum_{i=p}^{p+m-1}f_{i}^{*}g_{i+q-p}\equiv \sum_{i=p}^{p+m-1}f_{i}^{*}g_{i+d}\;\left(\text{here}\;d\equiv q-p\right)\\ & =\sum_{i=p}^{p+m-1}\left(s_{i+1,d}-s_{i,d}\right)\\ & =(s_{p+m,d}-s_{p+m-1,d})+(s_{p+m-1,d}-s_{p+m-2,d})+\ldots +(s_{p+1,d}-s_{p,d})\\ & =s_{p+m,d}-s_{p,d} \end{array}$$

## Appendix B

The 1D results presented are generated using the python code listed below. The “navigators.dat” file that is loaded from the main subroutine, is plain text. It contains three columns of length 128, separated from each other by “\t”. The first column is indexing from 1 to 128 into two other columns, the second column is the samples of the template, the third column is the samples of the navigator.

*#!* /*usr*/*bin*/*python*

**import** os

**import** numpy as np

**from** numpy **import** arange

**from** numpy **import** zeros

**from** numpy **import** absolute as **abs**

**from** numpy **import** square

**from** numpy **import** real

**from** numpy **import** sqrt

**from** numpy **import** exp

**from** numpy **import** concatenate as cat

**from** numpy **import** conjugate as conj

**from** numpy.fft **import** fft

**from** numpy.fft **import** ifft

**from** math **import** pi

**def** lewis_ccor (navigt, templt, N, Q, M, P):

 cc = zeros(P)   # *normalized cross-correlation*

 ns = zeros(N+1)  # *navigator sum*

 ns2 = zeros(N+1)  # *navigator sum of squares*

 **for** i **in range**(N):

  a = navigt [i]

  ns [i + 1] = a + ns [i]

  ns2 [i + 1] = a*a + ns2 [i]

 q = Q-1

 template = templt [q:q+M]

 ts = **sum**(template)      # *template sum*

 ts2 = **sum**(square(template))  # *template sum of squares*

 tm = ts/M         # *template mean*

 tv = ts2 − square(ts)/M    # *template variance*

 v1 = template − tm

 **for** i **in range**(P):

  k = i+M

  A = ns [k] − ns [i]

  C = ns2 [k] − ns2 [i]

  nm = A/M

  nv = C − A*A/M

  v2 = navigator [i:k] − nm

  numerator = **sum**(v1*v2)

  denominator = sqrt(tv*nv)

  cc [i] = numerator/denominator

 **return** cc

**def** luo_ccor (navigt, templt, N, Q, M, P):

 cc = zeros(P)  # *normalized cross-correlation*

 ns = zeros(N+1)  # *navigator sum*

 ns2 = zeros(N+1)  # *navigator sum of squares*

 tns = zeros((N+1,N))  # *template-navigator cross terms*

 **for** i **in range**(N):

  a = navigt [i]

  ns [i + 1] = a + ns [i]

  ns2 [i + 1] = a*a + ns2 [i]

  **for** d **in range**(N):

   k = (i+d)%N

   tns [i + 1] [d] = tns [i] [d] + templt [i]*navigt [k]

 q = Q-1

 template = templt [q:q+M]

 ts = **sum**(template)      # *template sum*

 ts2 = **sum**(square(template))  # *template sum of squares*

 tm = ts/M         # *template mean*

 tv = ts2 − square(ts)/M    # *template variance*

 **for** i **in range**(P):

  k = i+M

  A = ns [k] − ns [i]

  C = ns2 [k] − ns2 [i]

  nv = C − A*A/M

  d = (i-q)%N

  numerator = (tns [q+M,d] − tns [q,d]) − A*tm

  denominator = sqrt(tv*nv)

  cc [i] = numerator/denominator

 **return** cc

**def** template_functions (templt, kernel, N, Q, M, P):

 templt2 = square(**abs**(templt))

 tmp = ifft(fft(templt)*kernel)

 gc = tmp [**range**(P)]

 tmp = ifft(fft(templt2)*kernel)

 gg = real(tmp [**range**(P)])

 templt_padded = cat((templt [Q-1:Q+M-1],zeros(N-M)))

 FTpg = fft(templt_padded)/M

 **return** gc, gg, FTpg

**def** complex_ccor (navigt, gc, gg, kernel, FTpg, N, Q, M, P):

 navigt2 = square(**abs**(navigt))

 tmp = ifft(fft(navigt)*kernel)

 fc = tmp [**range**(P)]

 tmp = ifft(fft(navigt2)*kernel)

 ff = real(tmp [**range**(P)])

 FTnv = fft(navigt)

 tmp = fft(conj(FTnv)*FTpg)/N

 fgc = tmp [**range**(P)]

 q = Q-1

 gcq = gc [q]

 ggq = gg [q]

 numerator = real(fgc − conj(fc)*gcq)

 denominator = sqrt((ff − square(**abs**(fc)))*(ggq − square(**abs**(gcq))))

 **return** numerator/denominator

**if** __name__ == ‘__main__’:

 tx1 = 80

 tx2 = 106

 n = 128

 q = tx1

 m = tx2-tx1+1

 p = n-m+1

 A = np.fromfile(“navigators.dat”, sep=“\t”).reshape(n,3)

 template = []

 navigator = []

 **for** i **in range**(n):

  template = template + [A [i] [1]]

  navigator = navigator + [A [i] [2]]

 k = arange(1,n)

 kernel = (1.0/m) * ((exp(1 j * 2 * pi * m * k/n) − 1)/(exp(1 j * 2 * pi * k/n) − 1))

 kernel = cat(([1 + 1 j * 0.0], kernel))

 gc, gg, FTpg = template_functions(template, kernel, n, q, m, p)

 cc = complex_ccor(navigator, gc, gg, kernel, FTpg, n, q, m, p)

 lewis_cc = lewis_ccor(navigator, template, n, q, m, p)

 luo_cc = luo_ccor(navigator, template, n, q, m, p)

## Appendix C

For the 2D case we have

$$\begin{array}{ll} f_{i,i^{\prime }}= & \frac{1}{n\;n^{\prime }}\sum_{k=0}^{n-1}\sum_{k^{\prime }=0}^{n^{\prime }-1}\alpha_{k,k^{\prime }}\;e{\;}^{j2\pi \frac{ki}{n}}\;e{\;}^{j2\pi \frac{k^{\prime }i^{\prime }}{n^{\prime }}}\;0\leq i<n;\;0\leq i^{\prime }<n^{\prime }\\ & \Leftrightarrow \left\{f_{i,i^{\prime }}\right\}=if\;ft2\left(\left\{\alpha_{k,k^{\prime }}\right\}\right) \end{array}$$ (23)

$$\begin{array}{ll} \alpha_{k,k^{\prime }}= & \sum_{i=0}^{n-1}\sum_{i^{\prime }=0}^{n^{\prime }-1}f_{i,i^{\prime }}\;e{\;}^{-j2\pi \frac{ki}{n}}\;e{\;}^{-j2\pi \frac{k^{\prime }i^{\prime }}{n^{\prime }}}\;0\leq k<n;\;0\leq k^{\prime }<n^{\prime }\\ & \Leftrightarrow \left\{\alpha_{k,k^{\prime }}\right\}=f\;ft2\left(\left\{f_{i,i^{\prime }}\right\}\right) \end{array}$$ (24)

where *fft*2, *ifft*2 are respectively the 2D fast Fourier transform function and its inverse as defined in MATLAB. The 2D normalized cross-correlation is

$$\begin{array}{ll} \text{NCC} & =\frac{\frac{1}{mm^{\prime }}\sum_{i=p}^{p+m-1}\sum_{i^{\prime }=p^{\prime }}^{p^{\prime }+m^{\prime }-1}\left(f_{i,i^{\prime }}-\bar{f}\right)*\cdot (g_{i+q-p,i^{\prime }+q^{\prime }-p^{\prime }}-\bar{g})}{\sqrt{\frac{1}{mm^{\prime }}\sum_{i=p}^{p+m-1}\sum_{i^{\prime }=p^{\prime }}^{p^{\prime }+m^{\prime }-1}{\left|f_{i,i^{\prime }}-\bar{f}\right|}^{2}}\sqrt{\frac{1}{mm^{\prime }}\sum_{i=q}^{q+m-1}\sum_{i^{\prime }=q\prime }^{q\prime +m^{\prime }-1}{\left|g_{i,i^{\prime }}-\bar{g}\right|}^{2}}}\\ & \equiv \frac{\bar{f^{*}g}-{\left(\bar{f}\right)}^{*}\bar{g}}{\sqrt{\bar{{\left|f\right|}^{2}}-{\left|\bar{f}\right|}^{2}}\sqrt{\bar{{\left|g\right|}^{2}}-{\left|\bar{g}\right|}^{2}}} \end{array}$$ (25)

where *p*, *q* ∈ [0, *n* − *m* + 1]; *p*′, *q*′ ∈ [0, *n*′ − *m*′ + 1] and

$$\begin{array}{ll} \bar{f} & \equiv \frac{1}{mm^{\prime }}\sum_{i=p}^{p+m-1}\sum_{i^{\prime }=p^{\prime }}^{p^{\prime }+m^{\prime }-1}f_{i,i^{\prime }}\\ \bar{g} & \equiv \frac{1}{mm^{\prime }}\sum_{i=p}^{p+m-1}\sum_{i^{\prime }=p^{\prime }}^{p^{\prime }+m^{\prime }-1}g_{i+q-p,i^{\prime }+q^{\prime }-p^{\prime }}=\frac{1}{mm^{\prime }}\sum_{i=q}^{q+m-1}\sum_{i^{\prime }=q^{\prime }}^{q^{\prime }+m^{\prime }-1}g_{i,i^{\prime }}\\ \bar{{\left|f\right|}^{2}} & \equiv \frac{1}{mm^{\prime }}\sum_{i=p}^{p+m-1}\sum_{i^{\prime }=p^{\prime }}^{p^{\prime }+m^{\prime }-1}{\left|f_{i,i^{\prime }}\right|}^{2}\\ \bar{{\left|g\right|}^{2}} & \equiv \frac{1}{mm^{\prime }}\sum_{i=q}^{q+m-1}\sum_{i^{\prime }=q^{\prime }}^{q^{\prime }+m^{\prime }-1}{\left|g_{i,i^{\prime }}\right|}^{2}\\ \bar{f^{*}g} & \equiv \frac{1}{mm^{\prime }}\sum_{i=p}^{p+m-1}\sum_{i^{\prime }=p^{\prime }}^{p^{\prime }+m^{\prime }-1}f_{i,i^{\prime }}^{*}g_{i+q-p,i^{\prime }+q^{\prime }-p^{\prime }} \end{array}$$

Introducing

$$\begin{array}{c} \gamma_{k,k^{\prime }}\equiv \frac{1}{m\;m^{\prime }}\frac{\left(e{\;}^{j2\pi \frac{km}{n}}-1\right)}{\left(e{\;}^{j2\pi \frac{k}{n}}-1\right)}\frac{\left(e{\;}^{j2\pi \frac{k^{\prime }m^{\prime }}{n^{\prime }}}-1\right)}{\left(e{\;}^{j2\pi \frac{k^{\prime }}{n^{\prime }}}-1\right)} \end{array}$$ (26)

and

$$\begin{array}{c} h_{i,i^{\prime }}=\{\begin{array}{ll} g_{i+q,i^{\prime }+q^{\prime }}/\left(nm\right)/\left(n^{\prime }m^{\prime }\right) & 0\leq i<m;0\leq i^{\prime }<m^{\prime }\\ 0 & \text{otherwise} \end{array} \end{array}$$

we get

$$\bar{f}=if\;ft2\left(\left\{\alpha_{k,k^{\prime }}\gamma_{k,k^{\prime }}\right\}\right){|}_{p,p^{\prime }}$$ (27)

$$\bar{{\left|f\right|}^{2}}=ifft2\left(\left\{fft2\left(\left\{{\left|f\right|}_{i,i^{\prime }}^{2}\right\}\right){|}_{k,k^{\prime }}\gamma_{k,k^{\prime }}\right\}\right){|}_{p,p^{\prime }}$$ (28)

$$\bar{f^{*}g}=f\;ft2\left(\left\{\alpha_{k,k^{\prime }}^{*}f\;ft2\left(\left\{h_{i,i^{\prime }}\right\}\right){|}_{k,k^{\prime }}\right\}\right){|}_{p,p^{\prime }}$$ (29)

with similar terms for for $\bar{g}$ and $\bar{{\left|g\right|}^{2}}$.

The 2D result presented is generated with the 2D python code that can be found in the supplements. The two files “image1.dat” and “image2.dat” loaded from the main subroutine, are plain text. They contain the template and navigator image respectively. Both images are 512x512 “pixels” with real-value samples scaled from 0 to 255. The code runs equally well for other image sizes and images with complex-value samples.

## Supporting information

S1 File Supplement 1. (PDF)

S2 File Supplement 2. A zipped directory containing 6 files: (1)navigators.dat—1D real value measurements of length 128 samples, (2)complex_navigators.dat—1D complex value measurements of length 320 samples, (3)ncc1d.py—Python code used in the computation of 1D NCC, (4)image1.dat, (5)image2.dat—two separate 2D real value MRI images of abdomen, (6)ncc2d.py—Python code used in the computation of 2D NCC.
(ZIP)

S1 Fig Fig 1 illustrating the computations in Supplement 1. (EPS)
