# Supplementary material for: Computation of the normalized cross-correlation by fast Fourier transform
Source: PLoS One. 2018 Sep 20;13(9):e0203434. doi: 10.1371/journal.pone.0203434 (PMC6147431; doi:10.1371/journal.pone.0203434)
Supplement: S1 File — (PDF) [file pone.0203434.s001.pdf]

Supplement 1

An additional 1D case is investigated here to explore the possibility for a speedup in the computation that may result from different template and search region sizes. The file "complex_navigators.dat" contains five columns of length 320, separated from each other by "\t". The first column indexes the samples from 1 to 320. The second and third columns represent the real and imaginary values of the template's samples respectively. The fourth and the fifth column represent the real and imaginary values of the navigator's samples. The data file is created by using two separate 3D Fast Spin

S1 Fig 1. (**a**) Two intensity profiles of 320 samples each, a template of m=90 samples is selected on one of them (the heavy black line). (**b**) The template is slid along the other profile (the solid, red line) and the normalized cross-correlation (NCC) is computed for each possible position. NCC is then plotted as a function of the position of the left extremity of the template. (**c**) The template is drawn on top of the most similar chunk.

Echo (FSE) MRI measurements of the knee as provided by `http://mridata.org/fullysampled/knees`, measurement 2 and 6. The MRI kspace data are collected using exactly the same sequence parameters and an 8-channel acquisition coil. After transforming the kspace data to the image space by 3D IFFT, the image lines with coordinates x=1:320, y=206, and z=103 are extracted from either measurement. The lines of either measurement are then averaged over the coil's channels preserving their complex valuedness. One (averaged) line serves as the template, the other as the navigator. The sizes of the searched region are 30, 60, 90, 120, 150, and 180 samples all starting at sample q=100. The result for the searched region of 90 samples is presented graphically in S1 Fig 1. The execution times for two algorithms compared are tabulated in the S1 Table 1.

S1 Table 1. The time expendend in the computation of the NCC according to Lewis algorithm and the algorithm proposed in this paper.

# samples	method	mean time (μs)	std (μs)
30	Lewis, numerator frequency sp.	59.27	0.156
	Proposed method	77.40	0.003
60	Lewis, numerator frequency sp.	51.33	0.014
	Proposed method	78.30	0.218
90	Lewis, numerator frequency sp.	49.33	0.018
	Proposed method	74.90	0.024
120	Lewis, numerator frequency sp.	50.20	0.035
	Proposed method	73.10	0.033
150	Lewis, numerator frequency sp.	46.13	0.020
	Proposed method	68.20	0.003
180	Lewis, numerator frequency sp.	41.60	0.012
	Proposed method	67.20	0.001
